# Research on Forest Carbon Sequestration and Its Economic Valuation: A Case Study of the Zixi Mountain Nature Reserve, Chuxiong Prefecture

**DOI:** 10.3390/plants14172746

**Published:** 2025-09-02

**Authors:** Mengxue Pu, Shaohui Yang, Aimei Chen, Zhihua Deng

**Affiliations:** 1 College of Ecology and Environment, Southwest Forestry University, Kunming 650224, China; pmengx@swfu.edu.cn; 2 Department of Organizational, Southwest Forestry University, Kunming 650224, China; ysh@swfu.edu.cn; 3 School of Civil Engineering, Southwest Forestry University, Kunming 650224, China; chenaimei369@swfu.edu.cn

**Keywords:** carbon accounting, forest carbon stock, economic valuation, nature reserve

## Abstract

Improving the precision of forest vegetation carbon stock estimation is essential for scientifically evaluating its economic value and ecological benefits. This study aims to investigate the impact of different estimation methods on carbon stock and its economic value. Taking the forest vegetation of the Zixi Mountain Nature Reserve as the research object, the carbon stock of the arbor layer was estimated using four approaches: the variable biomass expansion factor method, the biomass expansion factor method, the volume conversion method, and the continuous function method of the biomass conversion factor. The carbon stocks of economic forests and shrublands were estimated using the average biomass method. The economic value of forest carbon storage was then evaluated through the market value method and the optimal pricing approach for forest carbon sinks. The results revealed no significant differences among the four estimation methods. The estimated arbor forest carbon stocks were 692,548.39 tC, 672,599.83 tC, 673,161.07 tC, and 400,369.17 tC, respectively, with an overall average of 609,669.62 tC. The biomass expansion factor method and the volume conversion method produce the most consistent results. The corresponding relative errors were 13.59%, 10.32%, 10.41%, and −34.33%, respectively. The continuous function method of the biomass conversion factor exhibited the greatest variability, mainly due to the influence of *Pinus yunnanensis* parameters. Among all methods, the biomass expansion factor method yielded the smallest relative error, making it the most suitable for estimating arbor carbon stocks in the study area. The total average economic value of forest carbon storage in the region was estimated at CNY 58.09 million. Among all forest types, *Pinus yunnanensis* contributed the highest carbon value, totaling CNY 50.48 million. In terms of economic value per unit area, *Pinus armandii* ranked first, with CNY 11,418.92 per hectare. Among different age groups of arbor forests, middle-aged stands had the highest carbon sequestration value, reaching CNY 36.87 million. Across all functional zones, the core zone showed the greatest economic value at CNY 29.34 million. Enhancing forest resource protection to maximize both carbon sink capacity and economic returns, as well as promoting forest carbon trading, can bring additional economic benefits to Southwest China while contributing to the achievement of the national “dual carbon” goals.

## 1. Introduction

Forests represent the largest carbon pool within terrestrial ecosystems [1,2], characterized by high carbon density and rapid carbon accumulation rates [3]. They play a vital role in mitigating the increase in atmospheric CO_2_ concentrations and alleviating global climate warming [4,5,6]. From a long-term perspective on addressing climate change, forests must enhance the durability of their carbon sequestration capacity [7] to ensure sustained and effective ecosystem services [8]. Although forests cover only about 30% of the Earth’s land surface, they account for approximately 80% of the CO_2_ absorbed by terrestrial ecosystems [9,10,11], making them the most efficient nature-based solution for enhancing global carbon sinks [12,13]. Forest carbon stocks not only reflect the structure and function of ecosystems but also represent a key parameter for assessing their carbon sequestration potential and overall carbon budget [14]. In response to the global challenge of climate change [7], countries have advanced climate governance through international agreements such as the United Nations Framework Convention on Climate Change and have promoted voluntary emission reduction initiatives and afforestation programs to enhance ecological sustainability [15]. China has pledged to peak CO_2_ emissions by 2030 and achieve carbon neutrality by 2060, with continued efforts to expand forest cover and enhance carbon sequestration capacity, thereby strengthening the climate-regulating function of forests [16,17,18]. Under the guidance of national strategies such as ecological civilization construction and the “carbon peak and carbon neutrality” goals, China’s industries are accelerating their transition toward green and low-carbon development. This transformation trend is not only an essential requirement for high-quality industrial development but also provides broad prospects and new opportunities for cultivating top-tier innovative talent in ecolog-ical and environmental disciplines.

Enhancing the reliability of regional forest carbon stock and economic value assessments is a critical issue in global change research and provides essential scientific support and a decision-making basis for formulating climate change mitigation strategies and forest carbon sink enhancement measures. Current methods for estimating forest carbon stocks can be categorized into three main types: (1) the biomass expansion factor method based on forest volume [19]; (2) model simulations using climate and forest habitat parameters [20]; and (3) remote sensing inversion and estimation techniques [21,22]. Differences exist among these methods in estimation results, necessitating the selection of the most appropriate method according to the characteristics of the study area to improve estimation precision. Several studies have explored this topic. Suárez-Fernández G E et al. [23] significantly improved forest carbon stock estimation accuracy by integrating multisource remote sensing data with random forest models. Liu K et al. [24] compared stepwise regression, support vector regression, and random forest methods for estimating forest aboveground biomass, finding that the random forest model using Landsat 5/TM data as input reliably estimated aboveground biomass. Pasalodos-Tato M et al. [25] quantified net carbon stocks of Andalusian forest arbors using the default method and the stock-change method, revealing that the default method produced higher estimates. Domke G M et al. [26] compared generalized allometric regression models and the component ratio method, observing that the latter yielded carbon stock estimates 16% lower on average, with significant differences between the two methods. Given China’s long-term continuous forest monitoring and rich data accumulation [27,28], biomass conversion remains the dominant approach for estimating forest vegetation carbon stocks [29]. Guo Z et al. [30] estimated biomass carbon stocks in China’s forests using the mean biomass density method, the mean ratio method, and the continuous biomass expansion factor method, finding differences among results, with the mean biomass density method yielding the highest estimates. Zhou Z et al. [22], based on plot survey data, used volume tables and mean form factor methods to calculate coniferous and broadleaf forest volume, and then explored the feasibility of the biomass expansion factor method, volume-derived biomass method, and biomass inventory method for estimating forest carbon stocks at small scales, concluding that there were no significant differences among these methods and that carbon stock estimates were not influenced by volume calculation methods. Li S et al. [31] compared the conversion factor method, the biomass expansion factor method, and the variable biomass expansion factor method, concluding that the conversion factor method is more suitable for estimating forest carbon stocks in Beijing. Variations in carbon stock estimates across regions and methods highlight the need to select suitable carbon estimation approaches based on study area characteristics.

Regarding carbon sink valuation, carbon has been widely assigned economic value [7]. Recently, scholars have investigated the economic value of forest carbon sinks at national, provincial, municipal, and county scales for various forest types [32,33,34,35,36]. These studies indicate that the economic value of forest carbon sinks primarily depends on the carbon sink volume and the unit price of carbon. Kazak J [37] estimated the carbon sink value of Polish forest ecosystems using a price of EUR 6.11 per ton of CO_2_; Raihan A [38], through a meta-analysis, found that the global economic value of forest carbon amounts to USD 2005 per hectare. As early as 1997, Solberg B [39] noted that Norway’s prevailing carbon price standard was equivalent to USD 49 per ton of CO_2_. Given the wide disparities in global carbon pricing, moderately slowing economic growth could facilitate the realization of forest carbon sinks’ economic potential [40]. China’s 14th Five-Year Plan (2021–2025) underscores the pivotal role of pricing mechanisms in enhancing ecosystem carbon sink capacity. Xu G et al. [41] demonstrated that with sustained decarbonization of the national energy mix, forest carbon sinks could play a critical role in offsetting emissions—enabling China to achieve carbon neutrality before 2060. Although carbon prices vary significantly across regions, a well-designed carbon pricing mechanism is essential for advancing the dual carbon goals.

Forest carbon sink accounting and carbon pricing can stimulate the transition of the socioeconomic system toward a low-carbon economy [42]. However, variations in data sources and accounting methods may introduce uncertainties in carbon stock estimation [43]. Against the backdrop of the dual carbon goals, precisely assessing forest carbon stocks and their economic value has become increasingly critical. Located at the gateway to Central Yunnan, the Chuxiong Yi Autonomous Prefecture possesses abundant forest resources but lags in economic development. Leveraging its natural forest endowment to develop a carbon sink economy not only supports the green and low-carbon transition but also helps alleviate emission reduction pressures from other sectors. As a major ecological barrier within the prefecture, the Zixi Mountain Nature Reserve harbors rich forest resources and substantial carbon stocks. This study, based on forest resource monitoring data from Zixi Mountain, explores the applicability and relative precision of multiple estimation methods for arbor layer carbon stocks at a small regional scale. The biomass–volume model remains a widely used approach for estimating forest biomass carbon stocks [44]. In this study, arbor carbon stocks were calculated using four methods: the variable bio-mass expansion factor method, the biomass expansion factor method, the volume conver-sion method, and the continuous function method of biomass conversion factor, based on which the most suitable estimation method was selected. Additionally, the average bio-mass method was applied to estimate the carbon stocks of economic forests and shrub-lands. Building upon the quantified forest carbon stocks in the study area, the economic value of carbon sequestration in forest vegetation was systematically assessed using both the market value method and the shadow price method for forest carbon sinks (optimal value). This approach not only provides a solid scientific reference for related research but also offers targeted theoretical guidance and practical case studies for cultivating green-oriented talent in the new era.

## 2. Materials and Methods

### 2.1. Study Area Overview

**Figure 1:**
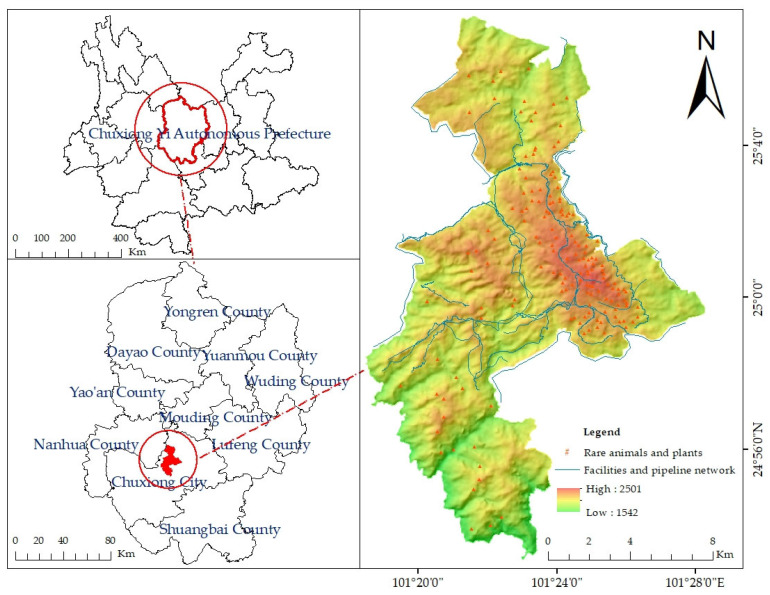
Location map of the study area.

The Zixi Mountain Provincial Nature Reserve is located in the central plateau region of Yunnan Province (Figure 1), positioned to the west of Chuxiong City, approximately 22 km from the urban center. It borders Nanhua County to the northwest and covers a total area of 16,000 hm^2^. The reserve lies between latitudes of 24°59′–25°04′ N and longitudes of 101°23′–101°28′ E, with an elevation range of 1542 to 2501 m above altitude. The area benefits from ample sunlight and a cool, humid climate typical of the northern subtropical zone, with distinct seasonal variation, abundant rainfall, and high humidity. The annual maximum average temperature reaches approximately 20 °C, with an average annual precipitation of 1000 mm and a relative humidity ranging from 80% to 85%. The region is endowed with exceptional natural resource conditions. Designated as a provincial-level nature reserve, Zixi Mountain primarily aims to protect representative subtropical semi-humid evergreen broadleaf forests in Central Yunnan, *Pinus yunnanensis* forest ecosystems, ancient *Camellia* sp., and a range of rare and endangered wild flora and fauna. It serves as a critical ecological barrier in Southwest China. The forest types within the reserve are mainly composed of water conservation forests, timber production forests, and natural conservation forests. This research focuses on seven dominant tree species within the area—*P. yunnanensis*, *Pinus armandii*, *Quercus* sp., *Eucalyptus robusta*, *Alnus cremastogyne*, *Cupressus funebris*, and *Keteleeria fortunei*—which collectively represent the main forest composition of the reserve. Among these, *P. yunnanensis* is the most widely distributed and exhibits robust growth throughout the study area. In addition to arbor forests, a small proportion of economic and shrub forests is also present.

### 2.2. Data Source

The data used in this study were derived from the 2019 forest resource monitoring records of Zixi Mountain, which were provided by the Chuxiong Prefecture Bureau of Natural Resources Protection. The survey was conducted at the forest compartment (sub-compartment) level and included information such as area, land use type, forest type, dominant tree species (or groups), age class, diameter at breast height (DBH), and standing volume. According to the statistical results, Zixi Mountain covers a total area of 16,000 hm^2^, which is subdivided into 114 forest compartments comprising 3031 sub-compartments. Among them, 2483 sub-compartments were classified as forest land, covering 15,044.71 hm^2^, including 94.19 hm^2^ of non-stocked forest land. This study focuses on three vegetation types—arbor forest, economic forest, and shrubland—representing a total surveyed area of 14,880.28 hm^2^ for carbon stock estimation.

### 2.3. Research Methods

#### 2.3.1. Forest Carbon Stock Estimation

(1) Estimation of arbor forest carbon stock

The volume of standing timber is a fundamental metric for determining forest carbon stocks, as it indirectly reflects the magnitude of carbon storage within a forest [45]. This study estimates the carbon stock of arbor forests based on the volume of standing timber. Four different volume-to-carbon conversion models were applied to calculate the carbon stock of arbor forests, and the resulting data were subjected to statistical analysis to identify the most appropriate model for estimating the carbon stock of the arbor forests in Zixi Mountain. All four methods utilized the same underlying data for calculation.

Method 1: variable biomass expansion factor method

The carbon stock of arbor forests is estimated using the age-class biomass expansion factor method [46] to enhance the precision of the estimation. Based on the volume of standing timber for each dominant tree species, the biomass of each species is calculated using age-class-specific biomass expansion factors. The carbon stock is then determined by converting the biomass into carbon content using the corresponding carbon content factor for each species. The parameters for each tree species are sourced from *The People’s Republic of China National Greenhouse Gas Inventory 2008* compiled by the Department of Climate Change, National Development and Reform Commission [47], as shown in Table 1. The calculation formula is as follows:

(1) $$C_{arbor\;forests}=\sum_{i=1}^{m}\sum_{j=1}^{n}V_{ij}\times D_{i}\times {BEF}_{ij}\times \left(1+R_{i}\right)\times {CF}_{i}$$

where *C_arbor forests_* is the total carbon stock of the arbor forest (tC); *V_ij_* is the standing timber volume of tree species *i* at age class *j* (m^3^); *D_i_* is the basic wood density of tree species *i* (t/m^3^); *BEF_ij_* is the biomass expansion factor for tree species *i* at age class *j*, which is the ratio of aboveground biomass to stem biomass, unitless; *R_ij_* is the root-to-shoot ratio for tree species *i* at age class *j*, which is the ratio of belowground biomass to aboveground biomass, unitless; *CF_i_* is the carbon content factor for the biomass of tree species *i*, unitless; *i* = 1, 2, …, *m*, represents tree species; and *j* = 1, 2, …, *n*, represents age classes.

Method 2: biomass expansion factor method

In this method, the biomass expansion factor approach [40] is applied without differentiating age classes. The parameters for each tree species use the overall values, as shown in Table 1. The formula is as follows:

(2) $$C_{arbor\;forests}=\sum_{i=1}^{m}V_{i}\times D_{i}\times {BEF}_{i}\times \left(1+R_{i}\right)\times {CF}_{i}$$

where *C_arbor forests_* is the total carbon stock of the arbor forest (tC); *V_i_* is the standing timber volume of tree species *i* (m^3^); *D_i_* is the basic wood density of tree species *i* (t/m^3^); *BEF_i_* is the biomass expansion factor for tree species *i*, which is the ratio of aboveground biomass to stem biomass, unitless; *R_i_* is the root-to-shoot ratio for tree species *i*, which is the ratio of belowground biomass to aboveground biomass, unitless; *CF_i_* is the carbon content factor for the biomass of tree species *i*, unitless; and *i* = 1, 2, …, *m*, represents tree species.

Method 3: volume conversion method

The carbon stock of a forest is the sum of the carbon stored in trees, understory vegetation, and soil in forest land. Since the carbon stock in understory vegetation and forest soil changes very little throughout the year or remains relatively stable [48,49], and since this study does not include herbaceous plants, litter, or forest soil carbon stock, the calculation for these components is excluded. Therefore, only the calculation for tree carbon stock is retained. The formula is as follows:

(3) C=Vδργ

where *C* is the carbon stock of the trees (tC); *V* is the timber volume (m^3^), excluding the volume of economic forests; *δ* is the biomass expansion coefficient, which is the coefficient used to convert forest volume into biomass, and it is generally taken as 1.90; *ρ* is the volumetric density, which is the coefficient used to convert biomass into dry biomass and is generally taken as 0.5; and *γ* is the carbon content factor, which is the coefficient used to convert dry biomass into carbon content, with a typical value of 0.5.

Method 4: continuous function method of biomass conversion factor

Fang et al. [50,51] compiled data from 758 research sets nationwide, relating biomass and stock volume, and classified China’s forest types into 21 categories. This research explored the relationship between biomass and stock volume, revealing a strong linear correlation between the two variables, which can be expressed as follows:

(4) B=aV+b

where *B* represents the biomass of the tree species (t); *a* and *b* are parameters that vary depending on the species; and *V* denotes the stock volume of the tree species (m^3^). The biomass for each species group, when multiplied by the corresponding carbon content factor (CF), provides the carbon stock (tC), as detailed in Table 1. The biomass equations for the dominant species are presented in Table 2.

(2) Estimation of carbon stock in economic forests

The economic forests in the Zixi Mountain Nature Reserve are primarily composed of fruit-bearing trees such as *Juglans regia*, *Prunus pseudocerasus*, *Prunus persica*, and *Pyrus*. As the database used in this study provides the area of economic forests but lacks stock volume data, it is not feasible to estimate biomass and carbon stock through traditional biomass–volume relationships. Therefore, drawing on the findings of Fang et al. [51], the average biomass of economic forests was set at 23.7 t/hm^2^, with a carbon content coefficient of 0.47. The total biomass was estimated by multiplying the average biomass per unit area by the total area of economic forests, and the carbon stock was subsequently calculated using the carbon content coefficient. The formula is as follows:

(5) $$C_{economic\;forests}=B_{economic}\times S_{economic}\times {CF}_{economic}$$

where *C_economic forests_* denotes the total carbon stock of the economic forests (tC); *B_economic_* is the average biomass per unit area of economic forests (t/hm^2^); *S_economic_* represents the total area of economic forests (hm^2^); and *CF_economic_* is the carbon content coefficient (dimensionless), with a value of 0.47.

(3) Estimation of carbon storage in shrub forests

This study estimated the total biomass of shrub forests by multiplying the mean biomass per unit area by the corresponding location. Subsequently, carbon storage was derived using the carbon fraction. The mean biomass value was adopted from Fang et al. [51], who provided reference data for estimating forest vegetation biomass across China. According to their findings, the average biomass of shrub forests is 19.76 t/hm^2^. The calculation formula is as follows:

(6) $$C_{shrub}=B_{shrub}\times S_{shrub}\times {CF}_{shrub}$$

where *C_shrub_* represents the total carbon stock of shrub forests (tC); *B_shrub_* denotes the average biomass per unit area (t/hm^2^); *S_shrub_* is the total area of shrubland (hm^2^); and *CF_shrub_* is the carbon fraction of shrub biomass, which is set to a default value of 0.47 (dimensionless).

#### 2.3.2. Data Analysis

The relative error (R_E_) was calculated using the following formula:

$$R_{E}=\frac{X-\mu }{\mu }\times 100\%$$

where *X* represents the estimated value, and *μ* denotes the average carbon stock obtained from the four calculation methods. A non-parametric test was conducted in SPSS 25.0 to assess the differences in carbon stock estimates among the four methods across seven tree species groups, with the significance level set at α = 0.05. Pairwise comparisons were carried out to evaluate the discrepancies among the methods. In conjunction with the relative error analysis, these comparisons were used to assess the performance of the four forests’ carbon stock estimation models. All visualizations were generated using ArcMap 10.8 and Origin 2021.

#### 2.3.3. Valuation of Forest Carbon Sequestration

As a unique type of public good, forest carbon sequestration is characterized by externalities, non-excludability, and non-rivalry [16,52]. It embodies multiple dimensions of value, which, in theory, can be quantified. The economic valuation of forest carbon sequestration involves monetizing the carbon stocks fixed by forests [49,53], thereby rendering the ecological benefits of forests more tangible. The economic value of forest carbon sequestration is calculated as the product of forest carbon stock and the unit price of carbon sequestration, which is expressed as follows:

(7) $$V=\frac{44}{12}\times C\times P$$

where *V* represents the economic value of forest carbon sequestration (CNY); *C* denotes the forest carbon stock (tC); *P* is the unit price of carbon sequestration (CNY/t CO_2_e); and 44/12 is the conversion factor from elemental carbon to carbon dioxide.

At present, China has not yet implemented a carbon tax system [54], and significant variations in carbon tax rates across countries make the carbon tax approach less applicable to the Chinese context. To ensure fairness and reliability in valuation, this study adopts both the market value method [55] and Zhang Y’s optimal (shadow) price approach [48] based on a review of the relevant literature. The arithmetic mean of the results obtained from these two methods is taken as the final carbon sequestration value to derive a more equitable and reasonable estimate of the economic value of forest carbon sequestration.

In 2019, the average transaction price across China’s eight carbon trading pilot regions—Beijing (83.27 CNY/t), Shanghai (41.70 CNY/t), Guangdong (18.96 CNY/t), Shenzhen (10.84 CNY/t), Hubei (29.50 CNY/t), Tianjin (14.00 CNY/t), Chongqing (6.91 CNY/t), and Fujian (16.89 CNY/t)—was 27.76 CNY/t CO_2_e. According to Zhang Ying’s research, the optimal price of forest carbon sequestration ranged between 10.11 and 15.17 USD/tC, which is equivalent to 2.76–4.14 USD/t CO_2_e when converted using the carbon-to-CO_2_ ratio. Using the lower bound of the optimal price range and the 2019 average exchange rate published by the China Foreign Exchange Trade Center (6.8985 CNY/USD), the unit price of forest carbon sequestration in 2019 is estimated at 19.02 CNY/t CO_2_e.

## 3. Results

### 3.1. Analysis of Carbon Stock Estimates Derived from Different Methodologies

**Figure 2:**
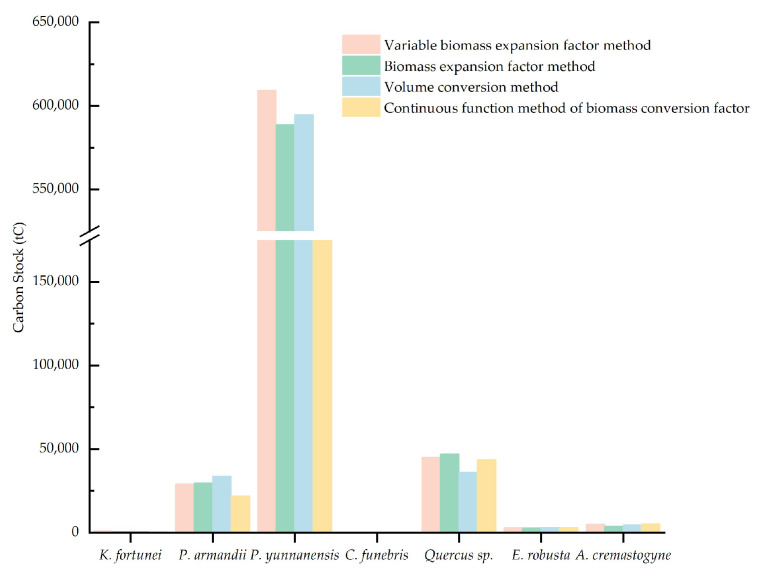
Carbon stock estimates for each tree species derived from four different methods.

**Figure 3:**
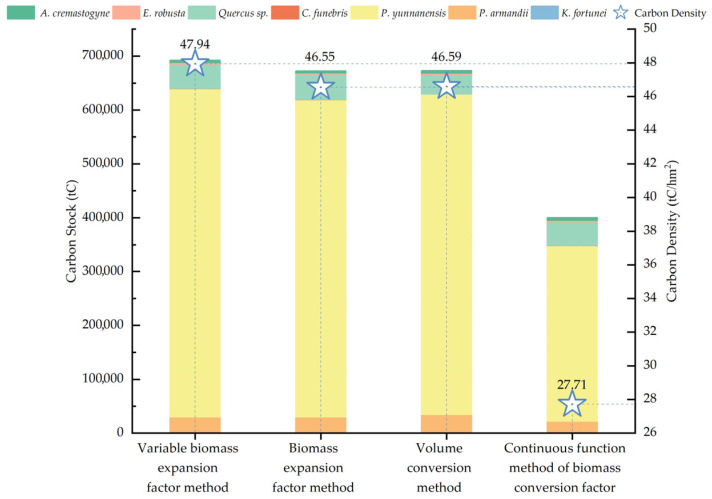
Estimated carbon stock and carbon density of arbor forests derived from four distinct calculation methods.

The carbon stocks of arbor forests estimated by four distinct methods are illustrated in Figure 2 and Figure 3. Except for *P. yunnanensis*, the estimates for different tree species derived from the four methods are generally consistent. However, the continuous function method of the biomass conversion factor produced a significantly lower estimate for *P. yunnanensis*, with a deviation of approximately 260,000 to 280,000 tC compared to the other three approaches. This deviation is primarily attributed to the low parameter values in the biomass regression equation for this species. Among the total carbon stock estimates for arbor forests, the variable biomass expansion factor method produced the highest value, reaching 692,548.39 tC. The biomass expansion factor method and the volume conversion method yielded relatively similar estimates of 672,599.83 tC and 673,161.07 tC, respectively. In contrast, the continuous function method of biomass conversion factor produced a significantly lower estimate of 400,369.17 tC—over 200,000 tC less than the other three—again largely due to the anomalously low estimate for *P. yunnanensis*. Nevertheless, non-parametric statistical analyses revealed no significant differences among the results obtained by the four methods. Pairwise comparisons using the Wilcoxon signed-rank test (Table 3) confirmed the absence of statistically significant differences. Moreover, Spearman’s rank correlation analysis demonstrated highly significant positive correlations (*p* < 0.01) among all pairs of methods (Table 4). These findings suggest that all four methods are statistically comparable and therefore suitable for estimating arbor forest carbon stocks at small spatial scales. The choice of method can thus be guided by the specific conditions of the study area and the practical feasibility of implementation.

**Figure 4:**
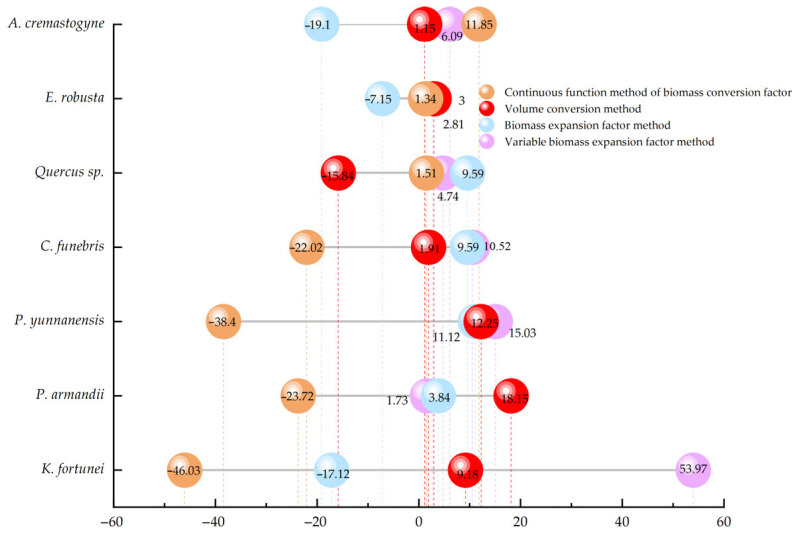
Comparison of relative errors among different tree species.

Currently, there is no universally accepted metric for evaluating the accuracy of carbon stock estimation methods [31,42], and the results of the aforementioned non-parametric tests do not allow for a definitive determination of the most reliable approach. Consequently, this research adopts the average value derived from the four methods as a reference baseline and calculates the relative error for each of the seven tree species groups. Based on the computational characteristics and the outcomes of the non-parametric analysis, we further identify the most suitable estimation method for the study area. As shown in Figure 4, the relative error analysis demonstrates that the variable biomass expansion factor method consistently yields positive deviations. Except for *Keteleeria fortunei*, the relative errors for other species are relatively concentrated, ranging from 1.73% to 15.03%. However, the relative error for *K. fortunei* is markedly higher, reaching 53.97%. The biomass expansion factor and volume conversion methods exhibit moderate error ranges of −19.10% to 9.59% and −15.84% to 18.15%, respectively. In contrast, the continuous function method of the biomass conversion factor is characterized by predominantly negative deviations, with relative errors ranging from −46.03% to 11.85%, again with *K. fortunei* showing the greatest deviation (−46.03%). In terms of overall error magnitude, the four methods can be ranked as follows: continuous function method of biomass conversion factor > variable biomass expansion factor method > volume conversion method > biomass expansion factor method. Table 5 summarizes the respective advantages and limitations of each estimation approach. Taking into account the *p*-values from non-parametric tests, the range of relative errors, and the methodological strengths and weaknesses—along with the availability of comprehensive local data—it is concluded that the biomass expansion factor method is the most appropriate for estimating carbon stocks in arbor forests within the study area. The other methods are retained for reference and comparative purposes only.

### 3.2. Current Status of Forest Resources and Carbon Stocks in the Nature Reserve

**Figure 5:**
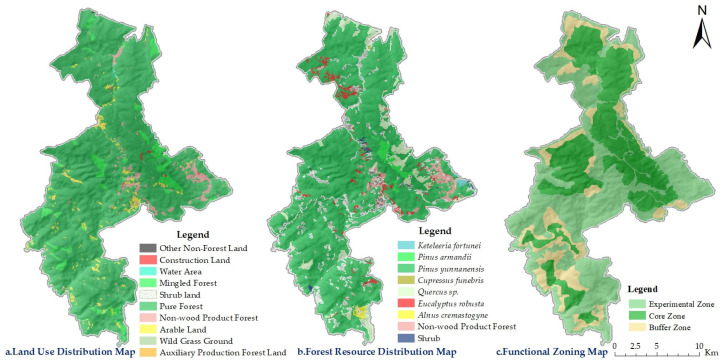
Land use, forest resource distribution, and functional zone in the Zixi Mountain Nature Reserve.

According to official records, the Zixi Mountain Nature Reserve spans an area of 16,000 hm^2^, of which 15,044.71 hm^2^ (94%) are classified as forestland. This includes 94.19 hm^2^ of non-stocked forestland. The remaining 955.21 hm^2^ (6%) are non-forested areas. The total forested area within the reserve amounts to 14,814.84 hm^2^, corresponding to a forest coverage rate of 93%. Among these, arbor forests occupy 14,447.58 hm^2^, with a total growing stock volume of 1417,181.20 m^3^. Additionally, there are 376.33 hm^2^ of economic forests, primarily comprising species such as *Pyrus*, *Prunus persica*, and *Juglans regia*, as well as 56.37 hm^2^ of shrubland. *P. yunnanensis* is the dominant species within the arbor forests, covering 12,789.08 hm^2^ (88.52% of the arbor forest area) and accounting for a timber volume of 1251,790.89 m^3^. The second most prevalent group is *Quercus* sp., occupying 932.09 hm^2^ (6.45%) with a stock volume of 76,041.34 m^3^. *C. funebris* forests are minimally distributed, covering only 6.73 hm^2^ (0.05%) and contributing a volume of 462.35 m^3^. The land use structure, forest resource distribution, and functional zoning within the study area are illustrated in Figure 5.

**Figure 6:**
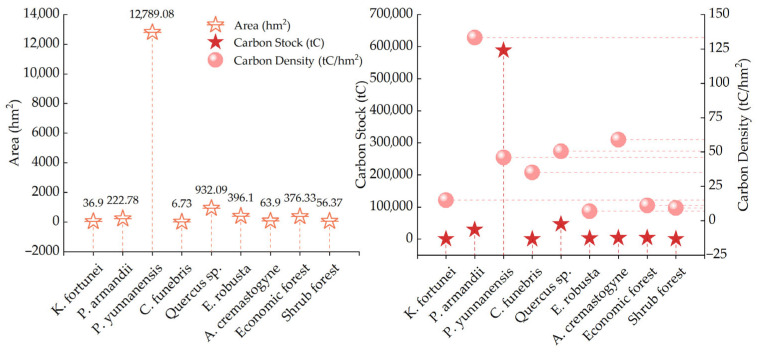
Area, carbon stock, and carbon density of different forest vegetation types.

In Section 3.1, the biomass expansion factor method was determined to be the most suitable approach for estimating the carbon stock of arbor forests in the study area. Consequently, the biomass expansion factor method was applied to calculate the carbon stock and carbon density of arbor forests, while the mean biomass method was used for estimating these metrics in economic forests and shrublands. The results are presented in Table 6. The total carbon stock of forest vegetation in the Zixi Mountain Provincial Nature Reserve reached 677,315.29 tC, with an average carbon density of 45.52 tC/hm^2^. Among these, economic forests accounted for 4191.94 tC, with a carbon density of 11.14 tC/hm^2^, and shrublands contributed 523.52 tC, with a density of 9.29 tC/hm^2^. Arbor forests dominated the carbon stock, contributing 672,599.83 t, which represents 99.30% of the total carbon stock, with an average carbon density of 46.55 t/hm^2^. Within the arbor forests, *P. yunnanensis* had the highest carbon stock, amounting to 588,618.02 tC, which accounted for 87.51% of the arbor forest carbon stock. This was followed by *Quercus* sp. and *P. armandii*, with carbon stocks of 47,029.75 tC and 29,661.94 tC, respectively. Combined, these two species accounted for 11.40% of the arbor forest carbon stock. Notably, although the area covered by *E. robusta* plantations exceeded that of *P. armandii* by 173.32 hm^2^, its carbon stock was only 2726.49 t—representing just 0.41%—highlighting a significant disparity in carbon storage capacity between these species. Among the seven dominant tree species, *C. funebris* had the lowest carbon stock, totaling just 236.16 tC. In terms of total vegetation carbon stock, the ranking was as follows: *P. yunnanensis* > *Quercus* sp. > *P. armandii* > economic forest > *A. cremastogyne* > *E. robusta* > *K. fortunei* > shrubland > *C. funebris*. As for carbon density, the order was *P. armandii* > *A. cremastogyne* > *Quercus* sp. > *P. yunnanensis* > *C. funebris* > *K. fortunei* > economic forest > shrubland > *E. robusta*. Detailed results are illustrated in Figure 6.

### 3.3. Analysis of Forest Carbon Sequestration Value

#### 3.3.1. Economic Value of Carbon Sequestration Across Forest Vegetation Types

Based on the calculated forest carbon stock, the economic value of carbon sequestration in the Zixi Mountain Provincial Nature Reserve was further evaluated, with the results summarized in Table 7 (For more detailed information, please refer to Table S1). The total economic value of forest carbon sequestration was estimated to range from CNY 47.23 million to CNY 68.94 million, with an average total value of CNY 58.09 million. Among different vegetation types, shrublands contribute only CNY 44,898.77, while economic forests contribute CNY 359,514.74. Together, these two forest types account for just 0.70% of the total carbon value. In contrast, the carbon storage value of arbor forests reaches CNY 57.68 million, representing a dominant 99.30% of the total. A closer examination of carbon values within arbor forests reveals that *P. yunnanensis* provided the highest contribution, with a carbon value of CNY 504.82 million, accounting for 87.51% of the total value in arbor forests. This is followed by *Quercus* sp., with a value of CNY 4.03 million (6.99%). In terms of both total carbon stock and economic value, *P. yunnanensis* shows a significant disparity compared to the other six forest types. This can be attributed primarily to its extensive distribution across the reserve—12,789.08 hm^2^—making up 79.93% of the study area, as it is the dominant afforestation species in Zixi Mountain. *C. funebris* species have the lowest economic value, contributing only CNY 20,254.08 (0.04%). This is consistent with their lowest carbon stock, a result of their limited distribution (only 6.73 hm^2^), with all stands being artificially planted and still in the juvenile growth stage, thus showing limited carbon sequestration capacity. In terms of carbon density and its corresponding economic value per unit area, *P. armandii* ranks first, with a carbon density of 133.14 tC/hm^2^ and an economic value of 11,418.92 CNY/hm^2^. *A. cremastogyne* follows with values of 59.06 tC/hm^2^ and 5064.99 CNY/hm^2^, closely followed by *Quercus* sp. (50.46 tC/hm^2^ and 4327.30 CNY/hm^2^). Despite *P. yunnanensis* having the highest total carbon value, it ranked fourth in terms of carbon density and value per hectare (46.03 tC/hm^2^ and 3947.26 CNY/hm^2^), reflecting the combined influence of factors such as forest area, growth performance, and species-specific biomass expansion factors.

**Figure 7:**
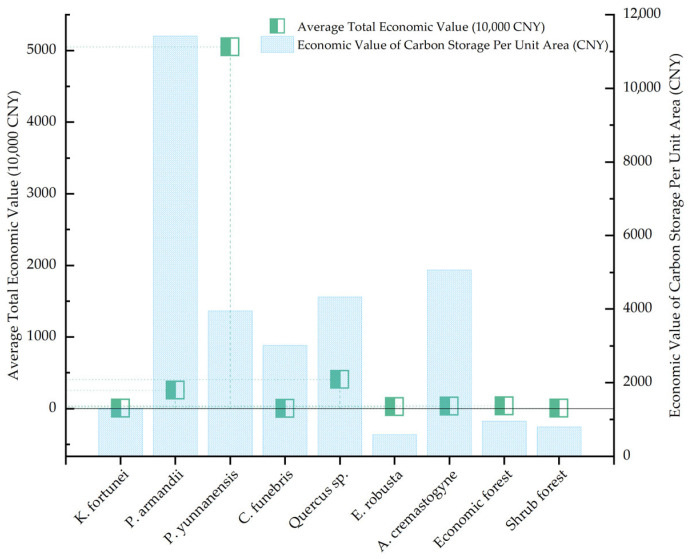
Economic value of carbon storage in different forest vegetation types.

The ranking of total carbon stock and its corresponding economic value among different forest vegetation types is as follows: *P. yunnanensis* forest > *Quercus* sp. forest > *P. armandii* forest > economic forest > *A. cremastogyne* forest > *K. fortunei* forest > *C. funebris* forest > *E. robusta* forest > shrubland. In terms of carbon density and carbon economic value per unit area, the order is *P. armandii* forest > *A. cremastogyne* forest > *Quercus* sp. forest > *P. yunnanensis* forest > *C. funebris* forest > *K. fortunei* forest > economic forest > shrubland > *E. robusta* forest (Figure 7). These results suggest that, in future forest stand regeneration and management efforts, priority should be given to tree species with higher carbon sequestration potential, such as *P. armandii*, *Quercus* sp., and *A. cremastogyne*. Meanwhile, the management and tending of the native species *P. yunnanensis* should remain a top priority, as it plays a pivotal role in regional afforestation efforts. Such strategies are crucial for expediting the achievement of “carbon neutrality” targets and promoting both sustainable forest ecosystem development and green economic growth.

#### 3.3.2. Carbon Sequestration Economic Value Across Age Groups of Arbor Forests

**Figure 8:**
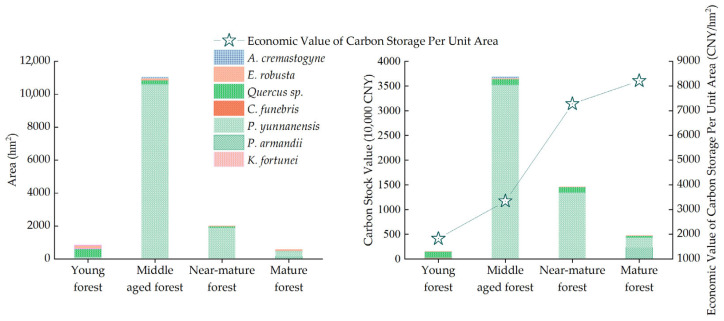
Area and economic value of carbon storage for different age groups of arbor forests.

According to the calculations, the total carbon sequestration economic value of arbor forests in the Zixi Mountain Nature Reserve ranges from CNY 46.91 to 68.46 million, with an average value of CNY 57.68 million. As shown in Figure 8, the economic value of carbon sequestration in arbor forests initially increases and then decreases with stand age. Middle-aged forests contribute the highest economic value, reaching CNY 36.87 million, accounting for 63.92% of the total. This is followed by near-mature forests at CNY 14.56 million (25.24%), mature forests at CNY 4.72 million (8.17%), and young forests, which contribute the least, at only CNY 1.53 million (2.67%). The ranking of carbon sequestration economic value across age classes is consistent with that of total carbon stock, following the order of middle-aged forest > near-mature forest > mature forest > young forest. In terms of carbon density and its economic value per unit area, the four age classes follow the trend of mature forest (95.77 tC/hm^2^, 8213.62 CNY/hm^2^) > near-mature forest (84.86 tC/hm^2^, 7278.26 CNY/hm^2^) > middle-aged forest (38.97 tC/hm^2^, 3342.30 CNY/hm^2^) > young forest (21.29 tC/hm^2^, 1825.82 CNY/hm^2^) (Figure 8, Table S2). These results indicate that both carbon density and the economic value of carbon stock per unit area increase with stand age.

#### 3.3.3. Carbon Sequestration Economic Value of Forest Vegetation Across Functional Zones

The nature reserve is divided into core, buffer, and experimental zones according to different levels of management requirements and ecological importance. As the name suggests, the core zone represents the essence of the reserve—it is the area with the most well-preserved natural ecosystems, where rare and endangered species are most concentrated and human disturbance is minimal. Access to this zone is strictly prohibited for all individuals and organizations. To shield the core zone from external interference, a buffer zone is established around it. This zone serves as a protective barrier and is designated exclusively for scientific observation and research purposes. Surrounding the buffer zone is the experimental zone, where a wider range of activities is permitted, including educational fieldwork, scientific research, ecotourism, and the domestication and breeding of rare and endangered wildlife species. The spatial layout of the functional zones within the Zixi Mountain Nature Reserve is illustrated in Figure 5.

**Figure 9:**
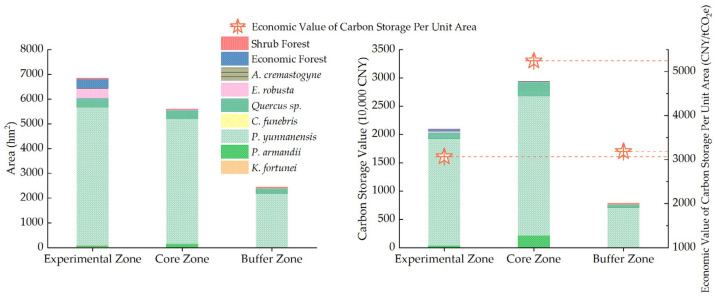
Forest vegetation area and carbon storage economic value across different functional zones.

Statistical analysis revealed a pronounced spatial heterogeneity in carbon stock and its corresponding economic value across the study area. As illustrated in Figure 5, both carbon stock and economic value exhibit a spatial distribution pattern characterized by higher values in the central and northern regions. Among the three functional zones, the core zone recorded the highest carbon stock, reaching 342,156.29 tC, with an associated economic value of CNY 29.34 million, accounting for 50.52% of the total carbon economic value. The experimental zone ranked second, with a carbon stock of 244,598.34 tC and an economic value of CNY 20.98 million, representing 36.11% of the total. The buffer zone had the lowest values, with a carbon stock of 90,560.65 tC and an economic value of CNY 7.77 million, accounting for only 13.37%. The spatial variation in carbon stock and its economic value is primarily attributed to differences in area and forest stand quality among the functional zones (Figure 9, Table S2). In terms of carbon density and carbon economic value per unit area, the core zone again ranked highest, with a carbon density of 61.14 tC/hm^2^ and a unit economic value of 5243.21 CNY/hm^2^. The buffer and experimental zones showed relatively similar values, with the buffer zone slightly outperforming the experimental zone. Specifically, the carbon density and economic value per unit area were 37.14 tC/hm^2^ and 3185.55 CNY/hm^2^ in the buffer zone and 35.73 tC/hm^2^ and 3064.44 CNY/hm^2^ in the experimental zone.

## 4. Discussion

### 4.1. Impact of Different Estimation Methods on Forest Carbon Stock Assessment

Different methods yield varying estimates of forest biomass and carbon stocks, and numerous studies have compared their results [22,31,42,56]. For example, a study on forest carbon stocks in southern Spain reported discrepancies between two estimation methods applied to the arbor layer [25]. In this study, we compared four estimation approaches and found that there are no significant differences among them, despite numerical biases. Aligning with the findings of Zhou Z [22], who reported similar results using three different estimation methods. In this research, the estimated arbor forest carbon stocks derived from the four methods were 692,548.39 tC (47.94 tC/hm^2^), 672,599.83 tC (46.55 tC/hm^2^), 673,161.07 tC (46.59 tC/hm^2^), and 400,369.17 tC (27.71 tC/hm^2^). The variable biomass expansion factor method produced the highest estimate, likely because it incorporates age-class-specific parameters that better reflect tree growth dynamics and thus provide more realistic results. The biomass expansion factor and volume conversion methods yielded highly similar estimates, as both rely on volume-to-biomass conversion, differing only in the parameters applied. Among them, the volume conversion method is the most straightforward to implement. In contrast, the continuous function method produced the lowest estimate, which may be attributed to the dominance of *P. yunnanensis* in the study area. Fang et al. [50] used only 12 samples to derive biomass parameters for this species, resulting in underestimated parameter values and, consequently, an underestimation of total carbon stock. Using the average of the four estimates as a reference, we conducted a relative error analysis. The continuous function method showed the largest negative deviation (−34.33%), indicating a severe underestimation. In comparison, the biomass expansion factor method had the smallest relative error (10.32%), followed closely by the volume conversion method (10.41%). These results demonstrate that the volume conversion method, biomass expansion factor method, and variable biomass expansion factor method are all well-suited for estimating the carbon stocks of arbor forests in Zixi Mountain. Among these, the volume conversion method is the simplest and most broadly applicable. However, based on a comprehensive assessment of relative accuracy and applicability, this study ultimately selected the biomass expansion factor method as the optimal approach. The total forest carbon stock of Zixi Mountain Provincial Nature Reserve was estimated at 677,315.29 tC, which is significantly higher than that of the Bandong Nature Reserve in Guangdong Province (289,471 tC) [57], but lower than that of the Miyaluo Nature Reserve in western Sichuan (5.86 TgC) [58]. This suggests that Zixi Mountain’s carbon stock falls within the mid-range among China’s nature reserves. Previous studies have shown that forest carbon stocks within protected areas are generally higher than those in unprotected regions [59], further underscoring the importance of ecological protection in enhancing forest carbon sink functions.

### 4.2. Influence of Different Estimation Methods on the Economic Value of Forest Carbon Sequestration

Currently, the most commonly employed methods for assessing the economic value of forest carbon sinks include the carbon tax method [60], afforestation cost method [61], artificial CO_2_ sequestration cost method [62], market value method [40], and the willingness-to-pay method [63]. Among them, the carbon tax method, afforestation cost method, and artificial CO_2_ sequestration cost method estimate value from a cost-based perspective, whereas the market value and willingness-to-pay methods approach valuation from an economic benefit standpoint. The differences in CO_2_ unit prices among these approaches directly result in variations in the estimated economic value of forest carbon stocks. Globally, over 70 carbon pricing schemes have been implemented, with China’s carbon prices notably lower than those in the European Union [64]. As summarized in Section 2.3.3, the average transaction prices from eight carbon trading pilot programs in China in 2019 have been summarized. Developing a nationwide carbon trading market in China not only allows western regions to leverage their natural resource advantages but also helps alleviate regional economic disparities and promote overall economic development [40]. As China’s carbon trading system continues to improve, this study estimates the carbon sequestration value of forest vegetation in the Zixi Mountain Nature Reserve using both the average CO_2_ transaction prices from the eight pilot markets and the optimal price of forest carbon sequestration. The results were CNY 68.94 million and CNY 47.24 million, respectively—a difference of 31.48% (CNY 21.71 million), with the latter being lower. Overall, the average total carbon sequestration value of the Zixi Mountain forest was CNY 58.09 million, which is lower than the carbon sequestration and oxygen release service value of the forest ecosystem in the Dianchi Lake Basin [65], and accounts for less than 1% of the carbon stock value of arbor forests in Mentougou District in 2020 [66]. Nevertheless, it exceeds the carbon sequestration value of Romania’s Retezat National Park in the same period (USD 170,607.03, which is approximately CNY 1.18 million) [35]. These findings further highlight the substantial carbon sink potential and ecological service value that the Zixi Mountain forest can provide for Chuxiong Prefecture and Yunnan Province.

The unit price of carbon is a key determinant in evaluating the economic value of forest carbon sequestration, and its variation is primarily influenced by differences in socioeconomic conditions, study regions, and methodological approaches. Shi X et al. [67], using the carbon tax method, estimated the carbon sink value of Chinese forest vegetation at CNY 406.83 and CNY 395.76 per ton in 2009 and 2013, respectively. Zhang Y [68], after adjusting for inflation and other factors, derived optimal carbon sink prices ranging from USD 10.11 to 15.17 per ton of carbon, corresponding to 8.11 CNY/tCO_2_ and 33.14 CNY/tCO_2_ for the years 2003 and 2008, respectively. Similarly, Roh T. W. [63] reported that Korean enterprises were willing to pay 5.45 USD/tCO_2_ domestically and 7.77 USD/tCO_2_ for international forest carbon sinks. In the United States, Plantinga A. J. [69] estimated CO_2_ prices in Wisconsin to range between USD 6.27 and 8.18 per ton. These findings collectively indicate that carbon pricing varies significantly across countries, regions, and methodological frameworks.

### 4.3. Comparative Analysis of Forest Carbon Sequestration Value by Forest Types, Age Classes, and Functional Zones

Overall, the forests within the reserve exhibit a significant carbon sequestration effect [70]. Among the various carbon pools, arbor forests account for the largest proportion and serve as a key carbon reservoir in forest ecosystems [71]. In the Zixi Mountain Nature Reserve, arbor forests contributed the highest carbon sequestration economic value, representing approximately 93% of the total. In contrast, economic forests and shrublands contributed only CNY 359,514.74 and CNY 44,898.77, respectively, together accounting for merely 0.7% of the total. This disparity primarily results from the dominance of arbor forests (14,447.58 hm^2^) in the reserve, while the areas of economic forests (376.33 hm^2^) and shrublands (56.37 hm^2^) are relatively limited. Further analysis revealed that among the seven arbor forest types, *P. yunnanensis* yielded the highest carbon sequestration economic value, reaching CNY 50.48 million. This species demonstrated a clear advantage over the other types due to its extensive distribution (12,789.08 hm^2^), accounting for 79.93% of the study area. Moreover, *P. yunnanensis* stands are primarily composed of middle-aged and nearly mature forests, which are known for their strong carbon fixation capacity. Previous studies have confirmed that older stands tend to store more carbon [72]. Additionally, as a pure coniferous forest, *P. yunnanensis* plantations in the reserve show no signs of pest or disease infestation, suggesting that the reserve has developed effective systems for pest control and disaster prevention. The stability of the forest ecosystem has also been reinforced by ecotourism revenue, which supports and enhances forest conservation efforts [70]. To further enhance the carbon sequestration capacity of the forest ecosystem and maximize its economic value, it is essential to optimize the management of mature and nearly mature stands to maintain their peak carbon sink potential. At the same time, greater attention should be paid to the cultivation and maintenance of young and middle-aged forests to promote their healthy development. As these stands mature, their carbon sequestration capacity through photosynthesis will gradually increase [73], thereby strengthening the forest’s overall carbon sink function and supporting the sustainable development of its ecological service value.

From the perspective of functional zoning, the core zone of the reserve holds the highest carbon stock and economic value, reaching 342,156.29 tC and CNY 29.34 million, respectively. Although the experimental zone covers a larger area (6845.49 hm^2^) and contains all dominant tree species along with most of the economic and shrub forests, its total carbon stock and economic value are lower than those of the core zone. This is primarily due to differences in stand age structure and the impact of human activities. Most *E. robusta* stands in the experimental zone are young, with low timber volume and limited carbon sequestration capacity. Furthermore, the area is open to tourists, and recreational activities may partially reduce the forest’s carbon sink function. In contrast, the core zone is dominated by middle- and near-mature stands of *P. yunnanensis*, *Quercus* sp., and *P. armandii* and serves as a key habitat for wildlife. As a strictly protected area with minimal human disturbance, favorable topography, and ample sunlight, trees in this zone exhibit vigorous growth and high biomass accumulation, resulting in superior carbon storage and economic value. Previous studies have shown that older forests with greater biodiversity tend to store more carbon [74,75,76], a conclusion further supported by the findings of this study. The notion that “lucid waters and lush mountains are invaluable assets” highlights that the value of ecological conservation lies not only in environmental benefits but also in its substantial economic potential.

## 5. Conclusions

This study compared the applicability and reliability of four carbon stock estimation methods in the Zixi Mountain Nature Reserve, finding that the variable biomass expansion factor method, biomass expansion factor method, and volume conversion method all reliably estimate the carbon stock of arbor forests, with the biomass expansion factor method providing a more moderate estimate. Based on this, the biomass expansion factor method was employed to calculate the carbon stock of arbor forests, while the average biomass method was used for economic forests and shrublands. The total forest vegetation carbon stock was estimated at 677,315.27 tC, with a carbon density of 45.52 tC/hm^2^. Using both the market value method and the optimal forest carbon sink pricing method to monetize carbon stock, the average total economic value was calculated to be CNY 58.09 million. The carbon stock economic value of arbor forests was the highest, accounting for 99.30% of the total carbon stock value within the study area. Among arbor forests, *P. yunnanensis* contributed the largest share, representing 87.51% of the carbon stock economic value, followed by *Quercus* sp. at 6.99%. Regarding forest age classes, middle-aged forests held the highest carbon stock economic value at 63.92%, followed by mature forests. Among functional zones, the core area exhibited the greatest carbon stock economic value, comprising 50.52% of the total, with the experimental area ranking second at 36.11%. In future management of the nature reserve, relevant personnel should strengthen ecological conservation awareness, enhance the prevention and control of harmful organisms, and rigorously prevent the invasion of alien species to minimize damage to the native ecosystem. Additionally, fire management must be continually reinforced, forest governance must be strictly enforced according to law, and all illegal activities harming wildlife and their habitats must be decisively combated. These measures will improve the stability and natural regeneration capacity of forest ecosystems, ensure the sustainable development of forest resources, and promote the construction of an ecological civilization characterized by harmonious coexistence between humans and nature. Ultimately, this will lay a firm foundation for the sustainable development of the ecological environment and the enhancement of human well-being.

This study has certain limitations, as it only accounts for the aboveground carbon stock of forest vegetation, excluding carbon stored in litter, herbaceous layers, and soil. Consequently, the carbon sequestration capacity of the Zixi Mountain forest ecosystem has been underestimated. Additionally, the economic value assessment does not account for the effects of inflation and exchange rate fluctuations. An accurate quantification of carbon stocks across all forest vegetation components is crucial for scientifically evaluating regional carbon sink capacity and its potential economic value. Future research should aim to improve the measurement of carbon stocks in all ecosystem carbon pools and enhance the precision of carbon stock estimation models to enable more accurate assessments of the carbon sequestration capacity and economic value of forest ecosystems. With the continued improvement of China’s carbon trading market and sustained efforts to strengthen forest conservation and sustainable management in the Zixi Mountain Nature Reserve, the generated green economic value is expected to increase significantly. This will also contribute to alleviating carbon reduction pressures in other sectors, thereby playing a vital role in China’s nationwide “dual carbon” goals.

## Figures and Tables

**Table 1:** Parameter information for each tree species [47].

Dominant Tree Species	Age Groups	BEF	R	D (t/m^3^)	CF
*K* *. fortunei*	Young forest	1.667	0.277	0.448	0.500
	Middle-aged forest	2.300	0.302		
	Near-mature forest	1.382	0.274		
	Mature forest	1.459	0.238		
	Total value	1.347	0.195		
*P* *. armandii*	Young forest	1.785	0.170	0.396	0.523
	Middle-aged forest	1.808	0.162		
	Near-mature forest	1.83	0.182		
	Mature forest	1.679	0.171		
	Total value	1.717	0.174		
*P* *. yunnanensis*	Young forest	1.619	0.146	0.483	0.511
	Middle-aged forest	1.837	0.143		
	Near-mature forest	1.333	0.238		
	Mature forest	1.585	0.190		
	Total value	1.585	0.202		
*C* *. funebris*	Young forest	1.732	0.220	0.478	0.510
	Middle-aged forest	1.847	0.218		
	Near-mature forest	1.497	0.233		
	Mature forest	1.233	0.329		
	Total value	1.535	0.365		
*Quercus* sp.	Young forest	1.355	0.292	0.676	0.500
	Middle-aged forest	1.380	0.260		
	Near-mature forest	1.327	0.275		
	Mature forest	1.360	0.410		
	Total value	1.587	0.153		
*E* *. robusta*	Young forest	1.263	0.221	0.578	0.525
	Middle-aged forest	1.297	0.219		
	Near-mature forest	1.178	0.221		
	Mature forest	1.165	0.181		
	Total value	1.151	0.226		
*A* *. cremastogyne*	Young forest	1.424	0.248	0.541	0.491
	Middle-aged forest	1.526	0.229		
	Near-mature forest	1.395	0.279		
	Mature forest	1.252	0.235		
	Total value	1.180	0.212		

**Table 2:** Biomass equations for dominant tree species [50].

Number	Dominant Tree Species (Group)	Biomass Estimation Model
1	*K* *. fortunei*	*B =* 0.4158*V +* 41.3318
2	*P* *. armandii*	*B =* 0.5856*V +* 18.7435
3	*P* *. yunnanensis*	*B =* 0.5101*V +* 1.0451
4	*C* *. funebris*	*B =* 0.6129*V +* 46.1451
5	*Quercus* sp.	*B =* 1.1453*V +* 8.5473
6	*E* *. robusta*	*B =* 0.8873*V +* 4.5539
7	*A* *. cremastogyne*	*B =* 1.0687*V +* 10.2370

**Table 3:** Results of the Wilcoxon signed-rank test for different methods.

Number	Comparison of Methods	*p*-Value
1	variable biomass expansion factor method vs. biomass expansion factor method	0.499
2	variable biomass expansion factor method vs. volume conversion method	0.176
3	variable biomass expansion factor method vs. continuous function method of biomass conversion factor	0.063
4	biomass expansion factor method vs. volume conversion method	0.310
5	biomass expansion factor method vs. continuous function method of biomass conversion factor	0.237
6	volume conversion method vs. continuous function method of biomass conversion factor	0.398

Note: “vs.” denotes comparison; the significance level is set at 0.05.

**Table 4:** Spearman rank correlation analysis of each method.

Method	Variable Biomass Expansion Factor Method	Biomass Expansion Factor Method	Volume Conversion Method	Continuous Function Method of Biomass Conversion Factor
variable biomass expansion factor method	1.000			
biomass expansion factor method	1.000 **	1.000		
volume conversion method	1.000 **	1.000 **	1.000	
continuous function method of biomass conversion factor	1.000 **	1.000 **	1.000 **	1.000

Note: ** indicates significance at the 0.01 level.

**Table 5:** Comparison of advantages and disadvantages of each carbon estimation method.

Method	Advantages	Disadvantages
Variable biomass expansion factor method	Incorporates age-class-specific expansion factors, providing a closer representation of actual forest conditions.	Requires high data accuracy and involves complex calculations.
Biomass expansion factor method	Widely applicable across various spatial scales; parameters are readily accessible.	Relatively high data accuracy requirements; assumes a constant biomass-to-volume ratio.
Volume conversion method	Straightforward and efficient; parameters are explicit and easily obtainable; relatively high accuracy.	Applies a uniform conversion coefficient across all forest types; does not account for stand factors such as age class, which may introduce error.
Continuous function method of biomass conversion factor	Integrates stand structure and age dynamics; suitable for various forest types.	Parameter estimation for some tree species lacks sufficient sample support.

**Table 6:** Carbon storage and carbon density of different vegetation types in the Zixi Mountain Nature Reserve.

Forest Land Types	Area (hm^2^)	Carbon Storage (tC)	Proportion of Carbon Stock (%)	Carbon Density (t/hm^2^)
arbor forest	14,447.58	672,599.83	99.30	46.55
economic forest	376.33	4191.94	0.62	11.14
shrub forest	56.37	523.52	0.08	9.29
total	14,880.28	677,315.29	100.00	45.52

**Table 7:** Economic value of carbon stocks in different forest vegetation types.

Forest Types		Carbon Stock Value (CNY 10,000)		Average Total Economic Value (CNY 10,000)	Value Proportion (%)
		Market Value Method (27.76 CNY/tCO_2_e)	Optimal Price (19.02 CNY/tCO_2_e)		
arbor forests	*K. fortune* forest	5.64	3.86	4.74	0.08
	*P. armandii* forest	301.92	206.86	254.39	4.38
	*P. yunnanensis* forest	5991.35	4105.02	5048.18	86.90
	*C. funebris* forest	2.40	1.65	2.03	0.03
	*Quercus* sp. forest	478.70	327.99	403.34	6.94
	*E. robusta* forest	27.75	19.01	23.38	0.40
	*A. cremastogyne* forest	38.41	26.32	32.37	0.56
	subtotal	6846.17	4690.71	5768.44	99.30
economic forests		42.67	29.23	35.95	0.62
shrub forests		5.33	3.65	4.49	0.08
total		6894.17	4723.60	5808.88	100

## Data Availability

The datasets used and/or analyzed during the current study are available from the corresponding author upon reasonable request.

## Supplementary Materials

The following supporting information can be downloaded at https://www.mdpi.com/article/10.3390/plants14172746/s1: Table S1: Summary of forest vegetation carbon stocks and their economic value in the Zixi Mountain Nature Reserve; Table S2: Carbon stock and economic value across age groups and functional zones.
