# Supplementary material for: Research on Forest Carbon Sequestration and Its Economic Valuation: A Case Study of the Zixi Mountain Nature Reserve, Chuxiong Prefecture
Source: Plants (Basel). 2025 Sep 2;14(17):2746. doi: 10.3390/plants14172746 (PMC12430626; doi:10.3390/plants14172746)
Supplement: Supplementary file 1 [file plants-14-02746-s001.zip › plants-3720164-supplementary.pdf]

Supplementary Materials

Table S1. Summary of forest vegetation carbon stocks and their economic value in the Zixi Mountain Nature Reserve.

Forest types		Area (hm²)	Carbon stock(tC)				Carbon sequestration economic value (CNY)		
			1	2	3	4	a	b	Mean
arbor forest	*K. fortunei*	36.9	1028.65	553.67	729.39	360.57	56356.10	38612.86	47484.48
	P. armandii	222.78	29060.79	29661.94	33748.77	21789.82	3019190.03	2068623.72	2543906.88
	P. yunnanensis	12789.08	609303.51	588618.02	594600.67	326295.33	59913466	41050220.58	50481843.29
	C. funebris	6.73	238.16	236.16	219.62	168.06	24038.19	16469.97	20254.08
	Quercus sp.	932.09	44949.56	47029.75	36119.64	43562.17	4787001.96	3279855.09	4033428.53
	E. robusta	396.1	3018.93	2726.50	3024.44	2975.63	277520.62	190145.61	233833.11
	A. cremastogyne	63.9	4948.80	3773.79	4718.54	5217.59	384121.56	263184.15	323652.86
	subtotal	14447.58	692548.39	672599.83	673161.07	400369.17	68461694.47	46907111.99	57684403.23
economic forest		376.33	4191.94				426683.59	292345.89	359514.74
shrub forest		56.37	523.52				53287.30	36510.25	44898.77
total		14880.28	677315.29				68941665.35	47235968.12	58088816.73

Notes：1- variable biomass expansion factor method；2- biomass expansion factor method；3- volume conversion method；4- continuous function method of biomass conversion factor； a- market value method; b- optimal (shadow) price approach.

Table S2. Carbon Stock and Economic Value across Age Groups and Functional Zones

Group		Area（hm²）	Carbon stock (tC)	Carbon sequestration economic value（CNY）		
				a	b	Mean
age groups	young forest	840.66	17896.90	1821665.57	1248129.65	1534897.61
	middle aged forest	11032.18	429937.63	43761918.64	29983850.6	36872884.62
	near-mature forest	2000.63	169782.37	17281581.06	11840622.18	14561101.62
	Mature forest	574.11	54982.93	5596529.19	3834509.56	4715519.37
functional zones	experimental zone	6845.49	244598.3436	24896850.07	17058288.48	20977569.27
	core zone	5596.66	342156.2891	34826948.14	23861979.60	29344463.87
	buffer zone	2438.13	90560.65448	9217867.15	6315700.04	7766783.60
